# Arthroscopically Assisted Cannulated Screw Fixation for Treating Type III Tibial Intercondylar Eminence Fractures: A Short-Term Retrospective Controlled Study

**DOI:** 10.3389/fsurg.2021.639270

**Published:** 2021-06-22

**Authors:** Chao Zheng, Huanli Han, Yujiang Cao

**Affiliations:** ^1^Department of Orthopaedics, Children's Hospital of Chongqing Medical University, Chongqing, China; ^2^Ministry of Education Key Laboratory of Child Development and Disorders, National Clinical Research Center for Child Health and Disorders, China International Science and Technology Cooperation Base of Child Development and Critical Disorders, Chongqing, China; ^3^Chongqing Key Laboratory of Pediatrics, Chongqing Engineering Research Center of Stem Cell Therapy, Children's Hospital of Chongqing Medical University, Chongqing, China; ^4^Department of Pediatric General Surgery and Liver Transplantation, Children's Hospital of Chongqing Medical University, Chongqing, China

**Keywords:** children, arthroscopy, tibial intercondylar eminence fractures, cannulated screws, treatment

## Abstract

**Background:** This study presents the clinical results from 22 children who underwent minimally invasive arthroscopically assisted screw fixation for the treatment of intercondylar eminence fractures.

**Methods:** We retrospectively analyzed the clinical data of 22 children (aged 7.5 to 13.5 years) with type III tibial intercondylar eminence fractures who were treated in our department from March 2007 to September 2019. According to the type of operation, the patients were divided into two groups: group A (*n* = 12) received arthroscopically assisted cannulated screw fixation, and group B (*n* = 10) received open reduction and cannulated screw internal fixation. Radiography scans, Lysholm scores, International Knee Documentation Committee (IKDC) 2,000 subjective scores, Tegner scores, range of motion (ROM) of the knee, the anterior drawer test (ADT), the Lachman test, and the pivot-shift test were used to evaluate the clinical efficacy.

**Results:** All 22 children were evaluated over a 12 to 58 month follow-up period (mean: 27.5 months). At the final exam, group A was significantly superior to group B in Lysholm scores (93.33 ± 3.55 vs. 86.20 ± 4.52), IKDC scores (92.06 ± 3.55 vs. 86.07 ± 5.81), and Tegner scores (7.75 ± 0.87 vs. 6.40 ± 0.52) and presented shorter operative times (25.42 ± 3.97 vs. 35.00 ± 5.27). The differences were statistically significant (*P* < 0.05). All the incisions healed primarily. No complications, such as fracture fragment displacement, delayed epiphyseal growth, or knee joint dysfunction, were observed. The drawer test, Lachman test, and pivot-shift test were negative for all patients.

**Conclusions:** Arthroscopically assisted cannulated screw fixation is effective and safe for the treatment of tibial intercondylar eminence fractures, providing excellent stability and quick recovery of joint function.

## Introduction

Tibial intercondylar eminence fractures are rare intra-articular fractures that primarily occur in children between 8 and 14 years of age (1). The incidence rate of such fractures is ~3/100,000 (2). Tibial intercondylar eminence fractures are located at the insertion of the anterior cruciate ligament (ACL) of the knee. Violent traction on the ACL is a common cause of tibial intercondylar eminence fractures in children (3). The ACL is prone to damage because the epiphyseal plate where the ACL attaches cannot provide sufficient force against the traction force of the ligament itself. Anatomic reduction of the fracture should be performed because intercondylar fractures are intra-articular fractures.

Clinically, fractures are often divided into three types (3): type I: no significant displacement of the fracture fragments; type II: displacement or slight titling of the first 1/3 of the anterior fracture fragment but normal positioning of the posterior fragment; and type III: complete displacement of the fracture fragments. Conservative treatment is often applied to treat type I fractures (4) and can achieve satisfactory results (5), while surgery is often required for type III fractures. With the development of endoscopic techniques, arthroscopic reduction with internal fixation has become a safe and prevalent technique used in the treatment of type III fractures (6). A recent systematic review showed the lack of evidence supporting the superiority of arthroscopic surgery relative to open surgery (7).

The ideal internal fixation device should have enough strength to meet early postoperative active rehabilitation. Cannulated screw fixation has been used widely in the treatment of tibial eminence fractures (8, 9). However, only a few studies have investigated this subject in skeletally immature patients (10). From March 2007 to September 2019, 22 patients with tibial intercondylar eminence fractures of type III were treated in our hospital. Among them, 12 cases were treated with arthroscopic-assisted cannulated screw fixation and 10 cases were treated by open reduction and internal fixation with cannulated screws. The curative effect is reported as follows.

## Materials and Methods

### General Data

We retrospectively analyzed the clinical data of 22 children with type III tibial intercondylar eminence avulsion fractures who were treated in our department from March 2007 to September 2019, and they included 11 males and 11 females. The fractures were classified according to the Meyers-McKeever classification (3). The children's ages ranged from 7.5 to 13.5 years old, with an average age of 11.1. The causes of the fractures included injury from sports (16 children) and car accidents (6 children). According to the type of operation, the patients were divided into two groups: group A (*n* = 12) received arthroscopically assisted cannulated screw fixation and group B (*n* = 10) received open reduction and cannulated screw internal fixation. The mean time from injury to surgical treatment was 6.2 days (between 2 and 11 days). The basic data of the patients are shown in Tables 1, 2.

**Table 1:** Patients' information (*n* = 34).

**Subject**	**Group 1**	**Group 2**
	***n* (%) or Mean ± SD (Range)**	***n* (%) or Mean ± SD (Range)**
*N*	24	10
Age (year)	10.94 ± 2.00 (8.0–14.0)	10.85 ± 1.53 (8.5–13.0)
BMI (Kg/m^2^)	19.71 ± 1.96 (16.5–23.1)	19.81 ± 2.17 (17.1–22.6)
**Sex (*****n*****)**		
Male	11 (45.8)	5 (50.0)
Female	13 (54.2)	5 (50.0)
**Type (*****n*****)**		
II	12 (50.0)	0 (0.0)
III	12 (50.0)	10 (100.0)
**Side (*****n*****)**		
Left	10 (41.7)	5 (50.0)
Right	14 (58.3)	5 (50.0)
Fracture duration (day)	6.00 ± 2.72 (2–11)	6.70 ± 2.31 (3–11)
Follow-up (month)	27.46 ± 11.80 (12–58)	27.1 ± 10.64 (16–46)

**Table 2:** Descriptive Date of Patients (*n* = 34).

**No**.	**Age**	**Gender**	**Side**	**Follow-up (month)**	**BMI**	**Type**	**Group**	**Fracture duration (day)**	**Op time (min)**	**Drawer**				**Lachmann**				**Pivot-shift**			
										**Preop**	**3 months**	**6 months**	**FFU**	**Preop**	**3 months**	**6 months**	**FFU**	**Preop**	**3 months**	**6 months**	**FFU**
1	9	Female	Left	34	17.7	II	A	10	20	+	0	0	0	+	0	0	0	0	0	0	0
2	13.5	Female	Left	20	21.8	II	A	5	25	+	0	0	0	+	0	0	0	0	0	0	0
3	9.5	Female	Right	30	18.2	II	A	3	25	+	0	0	0	+	0	0	0	0	0	0	0
4	11.5	Female	Left	19	18.6	II	A	7	20	+	0	0	0	+	0	0	0	0	0	0	0
5	9.5	Female	Right	31	17.6	II	A	2	25	+	0	0	0	+	0	0	0	0	0	0	0
6	12.5	Male	Right	24	21.6	II	A	8	25	+	0	0	0	+	0	0	0	0	0	0	0
7	7.5	Male	Left	29	16.5	II	A	9	20	+	0	0	0	+	0	0	0	0	0	0	0
8	9	Male	Right	31	18.2	II	A	10	15	+	0	0	0	+	0	0	0	0	0	0	0
9	14	Male	Left	17	22.4	II	A	6	15	+	0	0	0	+	0	0	0	0	0	0	0
10	10	Male	Right	50	19.8	II	A	5	15	+	0	0	0	+	0	0	0	0	0	0	0
11	9	Male	Left	32	19	II	A	5	20	+	0	0	0	+	0	0	0	0	0	0	0
12	12	Male	Right	27	20.5	II	A	4	15	+	0	0	0	+	0	0	0	0	0	0	0
13	7.5	Female	Left	18	16.7	III	A	7	25	++	0	0	0	++	0	0	0	+	0	0	0
14	10	Female	Right	12	18	III	A	3	20	++	0	0	0	++	0	0	0	+	0	0	0
15	12.5	Female	Right	28	20	III	A	2	25	+++	+	0	0	+++	+	0	0	+	0	0	0
16	13	Female	Left	19	22.7	III	A	3	25	+++	+	0	0	+++	+	0	0	+	0	0	0
17	12	Female	Right	45	19.8	III	A	5	30	+++	0	0	0	+++	0	0	0	+	0	0	0
18	8	Female	Right	40	17.1	III	A	7	30	++	0	0	0	++	0	0	0	+	0	0	0
19	11	Male	Left	58	20.5	III	A	8	25	++	0	0	0	++	0	0	0	++	0	0	0
20	13.5	Male	Right	13	20.8	III	A	10	20	+++	0	0	0	+++	0	0	0	+	0	0	0
21	12	Male	Left	28	23.1	III	A	6	30	+++	+	0	0	+++	+	0	0	+	0	0	0
22	13.5	Male	Right	14	21.9	III	A	11	30	+++	0	0	0	+++	0	0	0	+	0	0	0
23	11.5	Male	Right	25	20.8	III	A	5	25	+++	0	0	0	+++	0	0	0	+	0	0	0
24	11	Male	Right	15	19.8	III	A	3	20	++	0	0	0	++	0	0	0	++	0	0	0
25	8.5	Female	Right	16	17.4	III	B	3	30	++	0	0	0	++	0	0	0	+	0	0	0
26	10	Female	Left	46	17.1	III	B	8	35	++	0	0	0	++	0	0	0	+	0	0	0
27	12	Female	Right	17	17.7	III	B	4	35	+++	+	0	0	+++	+	0	0	+	0	0	0
28	11	Female	Left	28	19.4	III	B	5	40	+++	0	0	0	+++	0	0	0	+	0	0	0
29	9	Female	Right	36	18.4	III	B	7	40	++	0	0	0	++	0	0	0	+	0	0	0
30	11.5	Male	Left	16	22.6	III	B	11	45	+++	+	0	0	+++	+	0	0	++	0	0	0
31	12.5	Male	Right	40	22.5	III	B	8	30	+++	0	0	0	+++	0	0	0	+	0	0	0
32	11.5	Male	Left	21	21.8	III	B	7	35	++	0	0	0	++	0	0	0	++	0	0	0
33	9.5	Male	Right	22	19.5	III	B	8	30	++	0	0	0	++	0	0	0	+	0	0	0
34	13	Male	Left	29	21.7	III	B	6	30	+++	0	0	0	+++	0	0	0	+	0	0	0

*Group A, Arthroscopically assisted cannulated screw fixation; Group B, Open reduction and internal fixation with cannulated screws; Op time, Operation time; Preop, preoperative; FFU, Final follow-up; Drawer, anterior drawer test (ADT). The distance the tibia moved forward in the Drawer test and Lachmann test: ^+^1–5 mm; ^++^6–10 mm; ^+++^more than 10 mm. For Pivot-shift test: ^+^Grade I; ^++^Grade II; ^+++^Grade III*.

### Preoperative Preparation

The patients were fully evaluated before surgery. The evaluation included a detailed medical history, physical examination, and imaging examination. The anterior drawer test (ADT), Lachman test, and pivot-shift test were used to evaluate the stability of the knee joint (Table 2), the tests were done in the operating room under anesthesia on the operation day. Computed tomography (CT) and magnetic resonance imaging (MRI) can help determine the fracture classification, fracture fragment size, and combined injuries as well as the internal fixation device type and size. Preoperative MRI was performed in all patients, and injuries were identified.

### Surgical Instruments and Internal Fixation Devices

Arthroscopy system: A 4.0 mm × 30° arthroscope (Smith & Nephew, Inc.).

Cannulated screws: Cannulated fixation screws at 18–22 mm in size and 3–3.5 mm in diameter (NEWDEAL).

### Surgical Procedure

Under general anesthesia, the patient was placed in the supine position and the operative field was disinfected and draped. The tourniquet technique was then applied. For group A, an anterior medial and inferior minimal incision was performed below the patella and the structures in the joint cavity were examined via arthroscopy. Arthroscopic debridement was performed, and hematomas between the fracture fragments and the surrounding hyperplastic adhesion tissue were removed. An exploration hook was used to pull the fracture fragment with the ACL to the tibial plateau for reduction. After achieving satisfactory fracture reduction, a percutaneous 1.5 mm K-wire was obliquely inserted under 45° of flexion of the knee at the medial edge of the patella to temporarily fix the fracture fragments. During the operation, a C-arm X-ray machine was used to monitor the procedure. After achieving satisfactory reduction, a small incision was made at the site of the K-wire. The cannulated screw was placed over the K-wire under arthroscopic guidance to fix the fracture fragments. Then, the K-wire was removed and ACL tension was examined. A C-arm machine was used to observe the fracture reduction. A drainage tube was placed through the lateral incision of the joint cavity, and the incision was closed by one needle suture. After dressing the wound, the affected lower limb was immobilized at 15° of flexion with long leg plaster or a scaled brace. Patients in group B received an anterior medial and inferior minimal incision of 3–5 cm, and the fracture ends were exposed and cleared under direct vision. After manual reduction, the fracture was fixed with cannulated screws under the guidance of a 1.5 mm K-wire. Then, the K-wire was removed and ACL tension was examined. The C-arm was applied to observe the fracture reduction. The joint cavity was washed, and the incision was sutured. The postoperative treatment was the same as in group A.

### Postoperative Management

The joint cavity drainage tube was removed 2 to 3 days after surgery, and X-rays of the knee joint were repeated regularly. Rehabilitation training was carried out under the guidance of a doctor. The step-by-step principle was adopted to guide the children in performing isometric knee extension and ankle pump exercises to prevent muscle atrophy. Regular flexion and extension exercises were performed in a non-weight-bearing state starting 2 weeks after surgery. The flexion angle gradually increased according to fracture healing from 4 to 6 weeks after surgery. Generally, knee flexion can reach ~75–100° within 4–6 weeks while flexion can reach 120° within 6–8 weeks after surgery. The patients were initially allowed to ambulate with a supportive device and partially bear weight, and they were eventually permitted to ambulate without a supportive device and fully bear weight.

### Data Collection and Efficacy Evaluation

The anterior drawer test (ADT), Lachman test, and pivot-shift test were performed to test knee stability, and detailed preoperative and postoperative data were recorded (Table 2). The range of motion (ROM) of the knee in different periods was measured and recorded. Radiography scans, the Lysholm score (11), the International Knee Documentation Committee (IKDC) 2,000 subjective score (12), and the Tegner score (13) were used to evaluate the clinical efficacy.

### Statistical Analysis

Statistical analyses were performed using SPSS 26.0 (IBM, Armonk, NY, USA) statistical analysis software. The Mann-Whitney U test was used to compare the Lysholm scores, IKDC, Tegner score, and operation time between groups A and B (**Table 4**). GraphPad Prism 8.0.1 software was used to present the data.

## Results

No ACL rupture or meniscus tear was found on preoperative MRI examination in all patients. Some patients (No. 3, 4, 9, 10, 15 and 18) have long T2 signals of ACL, indicating edema and strain, but the ACL is intact. All 22 children completed a 12- to 58 month follow-up period (mean: 27.5 months). For the instability tests (Tables 2, 3), the preoperative front drawer test, Lachmann test and pivot-shift test were positive for all patients in the two groups. The positive signs of most patients disappeared at the 3 month follow-up after the operation, and all signs had disappeared by the 6 month follow-up. At the final follow-up, a KT-1000 system (MEDmetric, San Diego, USA) was used to evaluate the instability of the knee in some patients, and only 3 patients presented a displacement of 1–2 mm under knee flexion of 30 degrees. This finding indicates that both group A and group B achieved ideal knee stability after surgery. Table 3 also shows the recovery of ROM of the knee in the two groups, and it was more obvious in group A than group B at the 6 week follow-up but basically consistent between the groups at the last follow-up.

**Table 3:** Different evaluation scores at FFU (final follow-up), and changes of ROM (range of motion) (*n* = 34).

**No**.	**FFU**				**ROM**					**Complication**	**Secondary surgery**
	**Lysholm**	**IKDC**	**Tegner**	**KT-1000 S-to-S diff (mm)**	**Normal side**	**Injured side preop**	**Injured side postop 6 weeks**	**Injured side postop 12 weeks**	**Injured side postop FFU**		
1	97	97.7	10	0	0–145	0–95	10–95	0–145	0–145	None	–
2	97	97.7	9		0–150	0–90	5–100	0–145	0–145	None	–
3	100	98.9	10	0	0–155	10–95	10–90	0–150	0–155	None	–
4	95	96.6	9	0	0–145	0–95	5–95	0–145	0–145	None	–
5	93	92.0	8		0–150	0–100	10–95	0–145	0–145	None	–
6	97	100	10	0	0–145	0–90	0–100	0–150	0–150	None	–
7	95	94.2	9	0	0–150	10–90	0–100	0–150	0–150	None	–
8	93	93.1	9		0–155	0–90	10–90	0–150	0–150	None	–
9	95	90.8	9	0	0–150	0–95	5–95	0–150	0–150	None	–
10	95	92.0	8		0–145	0–95	5–95	0–145	0–145	None	–
11	97	96.6	9		0–150	0–90	10–95	0–145	0–150	None	–
12	95	97.7	9		0–145	0–95	5–100	0–145	0–145	None	–
13	97	96.6	8	0	0–150	10–85	5–100	0–150	0–150	None	–
14	95	93.1	9	0	0–155	15–80	0–100	0–150	0–150	None	–
15	97	94.2	8	0	0–145	10–80	0–100	0–145	0–145	None	–
16	93	86.2	7	0	0–150	20–70	10–90	0–150	0–150	None	–
17	98	96.6	8	0	0–155	15–75	5–95	0–150	0–155	None	–
18	90	93.1	8		0–145	20–60	5–95	0–140	0–140	None	–
19	93	90.8	9	0	0–150	15–70	10–95	0–150	0–150	None	–
20	97	94.2	8	0	0–145	15–80	0–100	0–145	0–145	None	–
21	90	92.0	7	2	0–150	20–80	10–90	0–150	0–150	None	Arthroscopic
22	93	90.8	8	0	0–155	10–75	5–95	0–155	0–155	None	–
23	90	92.0	7	0	0–150	15–80	5–100	0–145	0–145	None	–
24	87	85.1	6	0	0–150	10–80	0–95	0–150	0–150	None	–
25	87	87.4	7		0–155	20–70	20–85	0–140	0–140	None	–
26	85	80.5	6	0	0–145	15–75	10–90	0–130	0–135	None	–
27	90	94.2	7		0–150	20–60	15–85	0–150	0–150	None	–
28	87	82.8	6	0	0–145	15–70	20–70	0–140	0–145	None	–
29	81	81.6	6	0	0–150	20–70	20–80	0–125	0–135	None	–
30	80	79.3	7	2	0–155	15–75	20–75	0–145	0–150	None	–
31	90	90.8	6	1	0–145	20–60	25–80	0–145	0–145	None	–
32	92	92.0	7	0	0–150	15–70	10–90	0–130	0–140	None	–
33	80	80.1	6		0–155	15–80	25–80	0–120	0–140	None	–
34	90	92.0	6	0	0–145	20–80	20–85	0–140	0–145	None	–

*IKDC, International Knee Documentation Committee; S–to-S diff, Side-to-side difference; ROM, range of motion*.

**Figure 1:**
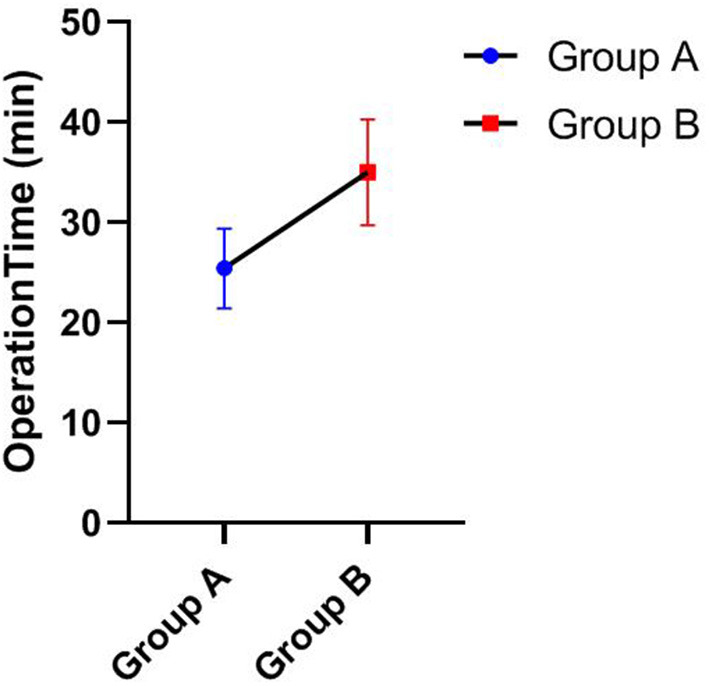
Operation time of different groups (Mean ± SD).

**Figure 2:**
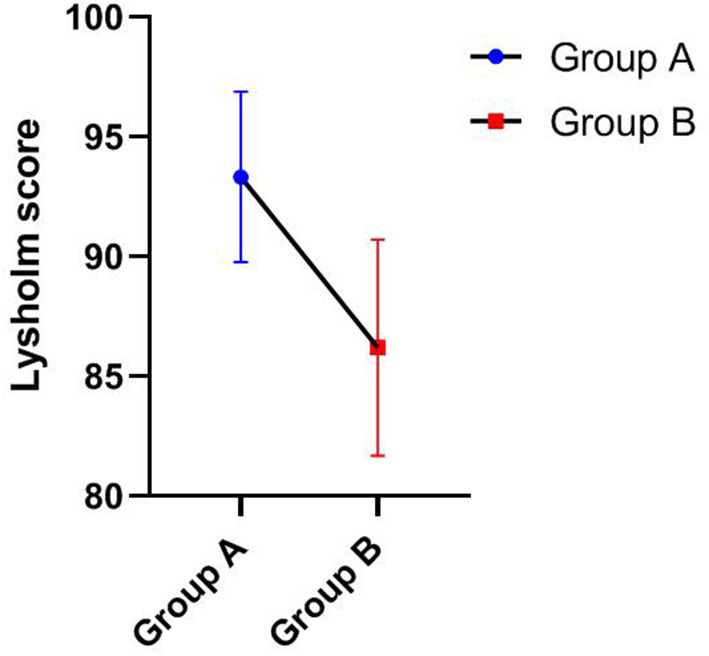
Outcome of Lysholm score of different groups at the final follow-up (Mean ± SD).

**Figure 3:**
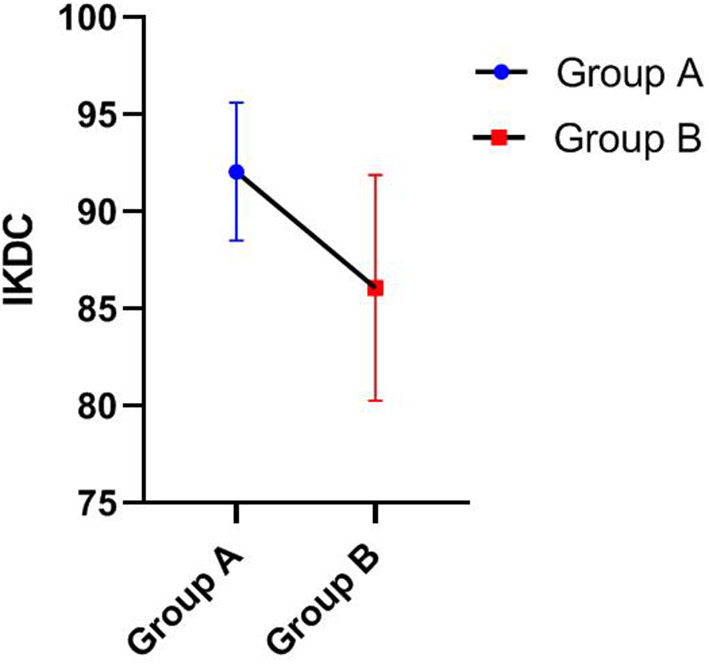
Outcome of IKDC of different groups at the final follow-up (Mean ± SD).

**Figure 4:**
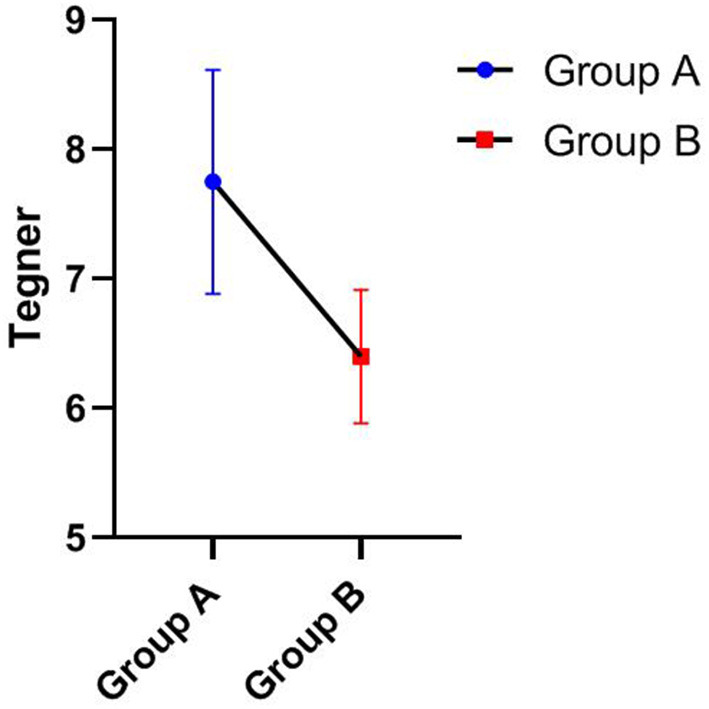
Outcome of Tegner score of different groups at the final follow-up (Mean ± SD).

As shown in Tables 3, 4, the Lysholm score, IKDC 2000 subjective score, and Tegner score were obtained for all patients at the last follow-up. The Mann-Whitney U test showed that group A was significantly superior to group B in Lysholm scores (93.33 ± 3.55 vs. 86.20 ± 4.52), IKDC scores (92.06 ± 3.55 vs. 86.07 ± 5.81), and Tegner scores (7.75 ± 0.87 vs. 6.40 ± 0.52), and the differences were statistically significant, *P* < 0.05. Figures 1–3 show the differences between the two groups. The operative time of the two groups was also recorded, and it was significantly shorter for group A than group B (25.42 ± 3.97 vs. 35.00 ± 5.27; *P* < 0.05), as shown in Table 3 and Figure 4. All instability tests and scoring statistics were performed by a single investigator.

**Table 4:** Statistical comparison of different evaluation scores between two groups.

	**Mean** **±** **SD**		**Mann-Whitney U test**	
	**Group A**	**Group B**	***Z***	***P***
Operation time (min)	22.71 ± 4.89	35.00 ± 5.27	−4.325	0.000
Lysholm	94.54 ± 3.06	86.20 ± 4.52	−4.111	0.000
IKDC	93.83 ± 3.71	86.07 ± 5.81	−3.307	0.001
Tegner	8.42 ± 1.02	6.40 ± 0.52	−4.148	0.000

*Mann-Whitney U test was performed, P < 0.05 indicating statistical significance*.

All incisions healed primarily, and complications, such as fracture displacement, delayed epiphyseal growth, intercondylar notch impingement, or knee joint dysfunction, were not reported. Six (27.3%) patients showed entrapment of the meniscus under a displaced tibial eminence fragment, and meniscus tears or ACL ruptures were not found. For secondary surgery, the parents of only one patient strongly requested removal of the internal fixation. We performed arthroscopic exploration and found that the intercondylar cartilage recovered well, with the cannulated screw tail completely covered by the cartilage. We abandoned the plan to remove the screws with the parents' consent.

**Figure 5:**
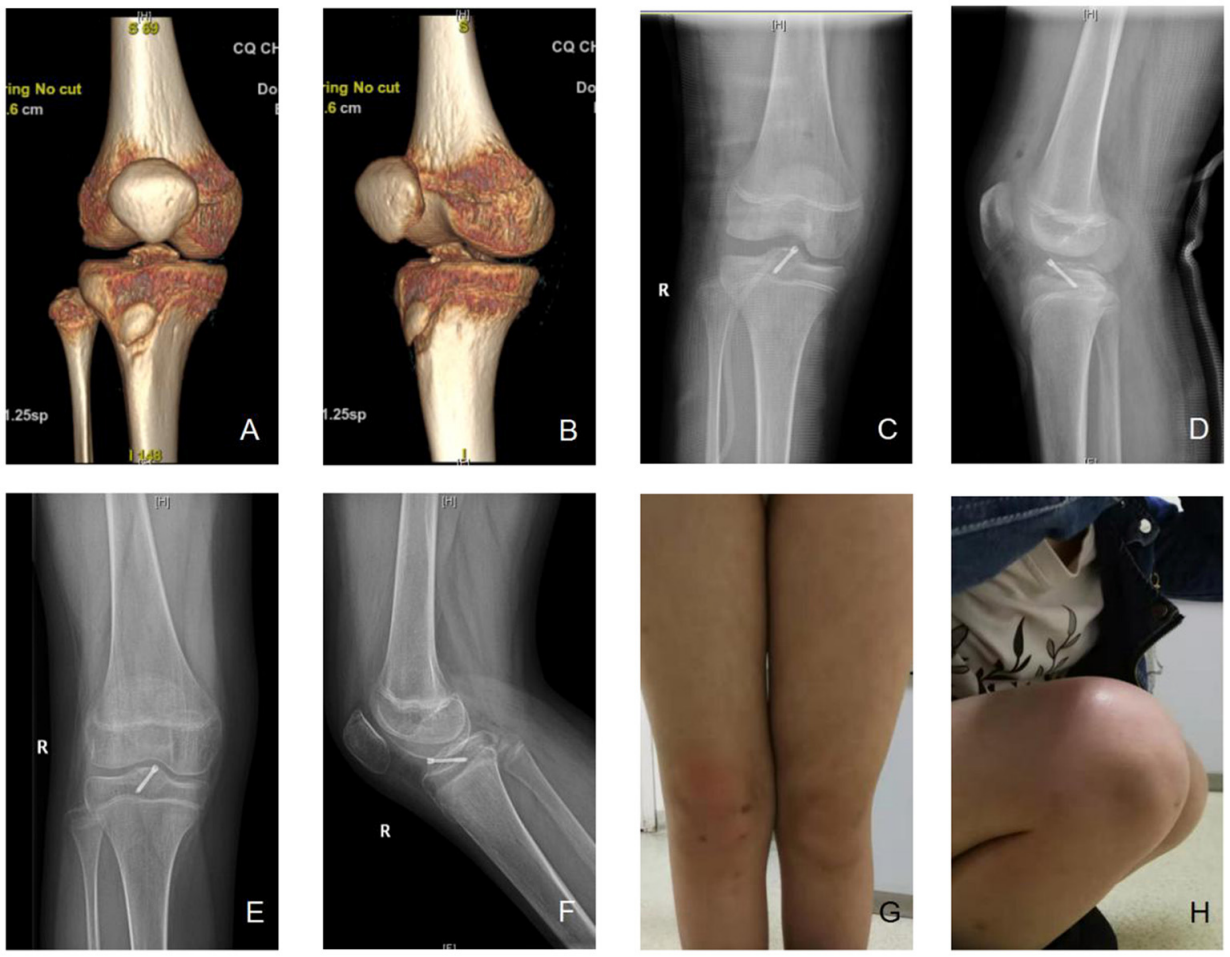
A typical case. **(A,B)** A 12-year-old female patient was diagnosed as tibial intercondylar eminence fractures, right, type III. Preoperative three-dimensional CT were shown. **(C,D)** Arthroscopic assisted internal fixation with hollow screws was applied. Postoperative X-ray showed good alignment of fracture. **(E,F)** 20 weeks after the operation, the X-ray showed that the fracture healed. **(G,H)** The appearance was good, and the flexion and extension activity of the knee was normal. The front drawer test, lackman test, and Pivot-shift test were negative, Lysholm score was 98, IKDC 96.6, Tegner score 8. No non-union, loosening of screw, or growth retardation of epiphyseal plate was observed.

A typical case is shown in Figure 5. The patient was a 12 year-old girl admitted due to joint swelling and pain in the right knee for 5 days after a fall. She was diagnosed with a type III tibial intercondylar eminence fracture. Figures 5A,B shows the three-dimensional CT images after admission. After admission, arthroscopically assisted cannulated screw fixation was performed. Figures 5C,D shows the radiography results on the first day after surgery. Postoperative follow-up was performed regularly in the orthopedic clinic, the fractures healed 8 weeks after surgery, and then the patients began functional exercises. Twenty weeks after the operation, good fracture healing and no internal fixation loosening, non-union, or delayed epiphyseal growth were observed as shown in Figures 5E,F, and the affected leg presents good appearance and function as shown in Figures 5G,H.

## Discussion

Tibial intercondylar eminence fractures in children are similar to ACL ruptures in adults (2), and they are primarily related to sports injuries (14, 15). Full skeletal maturity has not been reached in children, who show a thin and weak epiphysis that has less strength relative to a ligament, can only bear limited traction and is prone to avulsion fracture of the insertion rather than ligament body fracture. The main cause of the fractures in this study was sports injuries, which is consistent with results reported in the literature. The damage mechanism has been described in many studies (15–19) and is related to non-contact deceleration, jumping or twisting, and stretching, which involve changes in motion direction. Anatomic reduction of the fracture should be performed because tibial intercondylar eminence fractures in children are intra-articular fractures. Poor anatomic reduction can cause ACL relaxation, deformed fracture healing, secondary distal femoral or proximal tibial epiphysis injury, and meniscus injury, which can lead to knee osteoarthritis (16, 20) or joint fibrosis (21) in the long term. Therefore, early diagnosis and treatment of tibial intercondylar eminence fractures are important for children.

Traditional open reduction and internal fixation are important methods for the treatment of type III tibial intercondylar eminence fractures. However, this method has several disadvantages, including a longer incision, extensive surgical trauma, a longer hospital stay, slow recovery of knee joint function, and repeated knee joint stiffness after surgery. With the development and popularization of arthroscopic techniques, arthroscopic minimally invasive surgery for fracture reduction and internal fixation has basically overcome the above shortcomings and now represents the first-choice treatment of such fractures. Compared with open reduction surgery, arthroscopic minimally invasive surgery has the following obvious advantages (22–24): minimal trauma, clear intra-articular visualization for easy execution of the procedure, easy identification and treatment of other combined injuries in the joint, simple operation, reliable fragment reduction and fixation, fast postoperative knee function recovery, short disease course, and relatively lower hospitalization cost. In our study, the operation time of group A was significantly shorter than that of group B as shown in Table 3 and Figure 4. At the final follow-up, group A was significantly superior to group B in Lysholm score, IKDC score, and Tegner score, as shown in Figures 1–3. This finding indicates that arthroscopic techniques have the advantages of less damage and better recovery.

The most commonly used fixation devices for children with tibial intercondylar eminence fractures are screws, intraosseous sutures, Kirschner wires, and steel wires (23). The use of absorbable screws (9, 25) and suture anchors (26) has also been reported. The goal of treatment for children with intercondylar eminence fractures is to obtain anatomic reduction while maintaining proper tension of the ACL to achieve sufficient strength of the fracture fragments, which can facilitate early activities and prevent serious complications, such as joint stiffness. However, the choice of surgical approach remains controversial. During the same period, we used a variety of internal fixation devices to treat tibial intercondylar eminence fractures in children, such as Kirschner wires, absorbable cartilage screws (25), sutures, and cannulated screws. We found that although Kirschner wire fixation is simple and corresponds to minimal trauma and a short operative time, this technique results in unstable fixation, thereby precluding the performance of early functional exercises. Thus, joint stiffness is more likely. The exposed tail of the steel needle can increase the risk of knee infection. Although recent systematic reviews (5, 6) reported no significant difference in clinical outcomes between suture and screw fixation, intraosseous suture fixation requires intraoperative tunneling of the bone fragments and adjacent epiphysis, which increases the operative time and secondary damage to the tissue. Moreover, when the suture passes through the bone tunnel, the bone tissue is easily cut, and difficulties with suturing can increase the pressure applied to the bone fragment, which can easily lead to failed bone fragment fixation.

We concluded that the use of cannulated screws for the treatment of intercondylar eminence fractures in children has the following advantages. Cannulated screws can fix the fracture fragments through the guide needle, which is a relatively simple and faster operation that can be completed in approximately half an hour. Cannulated screw fixation is a reliable procedure, and subsequent displacement of the fracture fragments is uncommon. Moreover, this process is conducive to early knee joint function training and fast knee joint function recovery postoperatively. Senekovi et al. (27) reported good results when using cannulated screws with washers; however, washers may cause the screw head to bulge, thereby increasing the possibility of screw head bulging and extension impingement (10). In this study, we did not use washers and displacement of the fracture was not recorded. Arthroscopic surgery is associated with minimal trauma and pain that are easily tolerated. Only one cannulated screw provides for reliable and sufficient for fixation. Scholars (9, 10) have advocated the use of two screws to obtain better stability. Wiegand et al. (8) also obtained a good fixation effect with one Herbert screw, which was similar to the results of our study. We believe that the fracture fragment is small and that two screws increase the risk of fracture fragmentation.

We summarized the following surgical experience. At 45° of knee flexion, the Kirschner guide needle should be inserted from the medial oblique edge of the tibia. During surgery, the tissue, hematomas, and incarcerated adhesions in the fracture fragments should be removed. Effort should be directed toward achieving anatomical reduction. Kocher et al. (28) reported an ~54% incidence of entrapment of the meniscus or intermeniscus ligaments. A recent study from Korea showed an incidence of approximately 19%, which was similar to our results. The internal fixation device should be successfully placed during the first attempt; otherwise, the risk of cannulated screw loosening and epiphyseal and cartilage injury may be increased. Cannulated screws should not traverse the proximal tibial growth plate in skeletally immature patients to avoid the possibility of partial physeal arrest. In this study, all patients were skeletally immature at the time of surgery and no disturbance of physeal growth was observed at the final follow-up. Due to the biocompatibility of cannulated screws, a second surgical removal of the screws is generally not required. If screw head impingement occurs, physeal tethering is observed in a growing number of patients, or pain or other intolerable conditions exist, a second operation can be considered to remove the screw (29); however, none of these conditions were reported in our study.

The most common complications reported were anterior instability, stiffness, and pain with knee extension (30). Park et al. (30) suggested that an increase of more than 50% in the initial length of ACL fibers provided clear evidence of ACL laxity, which may result in anterior instability. In our study, the preoperative front drawer test, Lachmann test and pivot-shift test were positive in all patients in the two groups, positive signs in most patients disappeared at the 3 month follow-up after the operation, and all signs disappeared during the 6 month follow-up. Although long T2 signals of ACL were found on preoperative MRI in some patients, suggesting edema and strain, the stability was satisfactory at the last follow-up. Only 3 patients were found to have a displacement of 1–2 mm under knee flexion of 30 degrees by the KT-1000 system. The cannulated screw is suggested to have good stability and strength. Meanwhile, the metaphyseal strength is less than that of ligament tissue and the tensile capacity is weak in children at skeletally immature age; therefore, fracture is more likely to occur than ACL elongation. Fabricant et al. (31) and Shin et al. (10) reported a 31 and 4% the incidence of stiffness, respectively, which may be related to the different methods of brace fixation and functional training after operation. In a small sample study, Wiegand et al. (8) reported detailed changes in the ROM recovery process, and all patients returned to normal within 12 weeks postoperatively, which was similar to our results. As shown in Table 3, the recovery of ROM of group A was more obvious than that of group B at the 6 week follow-up but basically the same at the last follow-up. This finding indicates that in terms of ROM recovery, although the final outcomes of the two groups were similar, arthroscopic technology has more advantages in the process of ROM recovery. We suggest that regular flexion and extension exercises should be started in a non-weight-bearing state 2 weeks after surgery and the knee flexion angle should be gradually increased 4–6 weeks after surgery.

This study presented certain limitations. First, the follow-up time was short and some patients were not followed to a skeletally mature age. Second, the treatment only included arthroscopically assisted cannulated screw fixation and open reduction and cannulated screw internal fixation and did not involve the comparison of different internal fixation methods, such as screws, sutures, Kirschner wires and steel wires. Third, this study was limited to type III tibial intercondylar ridge fractures in children, and control studies were not conducted on other types of fractures. Fourth, group A was significantly superior to group B in Lysholm scores, IKDC scores, and Tegner scores, while group B included patients who complained about pain or instability. However, due to age and cognitive level, their experience may not be completely objective. Some patients may not participate in or may dislike some sports items on the Tegner rating scale; thus, a certain bias may affect the accuracy. Therefore, the clinical efficacy and related complications still need to be further confirmed by prospective randomized controlled trials.

In summary, arthroscopically assisted cannulated screw fixation is effective and safe for the treatment of tibial intercondylar eminence fractures, providing excellent stability and quick recovery of joint function. Extending the method's application to the treatment of intercondylar eminence fractures in children is worthwhile.

## Data Availability Statement

The original contributions presented in the study are included in the article/supplementary material, further inquiries can be directed to the corresponding author/s.

## Ethics Statement

This study was approved and supervised by the Institutional Review Board of Children's Hospital of Chonging Medical University. Written informed consent to participate in this study was provided by the participants' illegal guardian/next of kin. Written informed consent was obtained from the individual(s), and minor(s)' illegal guardian/next of kin, for the publication of any potentially identifiable images or data included in this article.

## Author Contributions

CZ analyzed the data and wrote the paper. HLH performed the statistical measurements. YJC designed and evaluated the manuscript. All authors contributed to the article and approved the submitted version.

## Conflict of Interest

The authors declare that the research was conducted in the absence of any commercial or financial relationships that could be construed as a potential conflict of interest.

## Abbreviations

ACL: Anterior cruciate ligament
CT: Computed tomography
MRI: magnetic resonance imaging
ADT: Anterior Drawer Test
IKDC: International Knee Documentation Committee.
